# The association between socioeconomic status and traditional chinese medicine use among children in Taiwan

**DOI:** 10.1186/1472-6963-12-27

**Published:** 2012-02-01

**Authors:** Chun-Chuan Shih, Chien-Chang Liao, Yi-Chang Su, Tsu F Yeh, Jaung-Geng Lin

**Affiliations:** 1 The School of Chinese Medicine for Post-Baccalaureate, I-Shou University, Kaohsiung County 82445, Taiwan; 2 Graduate Institute of Chinese Medicine, China Medical University, Taichung 404, Taiwan; 3 Department of Anesthesiology, Taipei Medical University Hospital, Taipei 110, Taiwan; 4 Health Policy Research Center, Taipei Medical University Hospital, Taipei 110, Taiwan; 5 Management Office for Health Data, China Medical University Hospital, Taichung 404, Taiwan; 6 School of Medicine, College of Medicine, China Medical University, Taichung 404, Taiwan; 7 Taipei Chinese Medical Association, Taipei 100, Taiwan

## Abstract

**Background:**

Traditional Chinese medicine (TCM) utilization is common in Asian countries. Limited studies are available on the socioeconomic status (SES) associated with TCM use among the pediatric population. We report on the association between SES and TCM use among children and adolescents in Taiwan.

**Methods:**

A National Health Interview Survey was conducted in Taiwan in 2001 that included 5,971 children and adolescents. We assessed the children's SES using the head of household's education, occupation and income. This information was used to calculate pediatric SES scores, which in turn were divided into quartiles. Children and adolescents who visited TCM in the past month were defined as TCM users.

**Results:**

Compared to children in the second SES quartile, children in the fourth SES quartile had a higher average number of TCM visits (0.12 vs. 0.06 visits, p = 0.027) and higher TCM use prevalence (5.0% vs. 3.6%, p = 0.024) within the past month. The adjusted odds ratio (OR) for TCM use was higher for children in the fourth SES quartile than for those in the first SES quartile (OR 1.49; 95% confidence interval [CI] 1.02-2.17). The corresponding OR was 2.17 for girls (95% CI 1.24-3.78). The highest-SES girls (aged 10-18 years) were most likely to visit TCM practices (OR 2.47; 95% CI 1.25-4.90).

**Conclusions:**

Children and adolescents with high SES were more likely to use TCM and especially girls aged 10-18 years. Our findings point to the high use of complementary and alternative medicine among children and adolescents.

## Background

Complementary and alternative medicine (CAM) is an increasingly popular therapeutic mode among adults and children all over the world [1-6]. CAM use and expenditures among adults in the US increased substantially between 1990 and 1997. This phenomenon has been attributed primarily to an increase in the proportion of the population seeking alternative therapies rather than to an increase in the number of visits per patient [1]. In 1997 it was estimated that 42% of US adults used CAM. At 629 million visits, CAM use by Americans in 1997 exceeded even the total number of visits to primary care physicians [1]. The prevalence of CAM use remained stable from 1997 to 2002 [7]. About 40% of parents in the US were CAM users during this time, whereas 21% had treated their child with CAM over the preceding year [5].

The National Centre for Complementary and Alternative Medicine at the National Institutes of Health defined CAM as a diverse group of medical and health systems, practices and products that are not presently considered to be part of conventional allopathic medicine (AM) [8]. In Taiwan, traditional Chinese medicine (TCM) is legal and like AM, is covered by the National Health Insurance. TCM includes acupuncture, herbal medicine, moxi-bustion, Tuina, Baguan and their techniques. TCM use is common among Koreans and people in Taiwan [9-14]. Even among white-collar Caucasians in Taiwan, the prevalence of TCM use has been reported to be as high as 45% [15].

While TCM and CAM are commonly used in Taiwan [10-13] and among immigrant Chinese populations in Canada and the United States [16,17], their use in western countries is increasing [2,3,7]. It was estimated in 2005 that about 72 million US adults had used CAM within the past year [7]. With such large numbers of people using CAM, the context surrounding its use should not be ignored.

CAM use for children with special health care needs is also common (64%), especially among children with chronic illnesses or disabilities in the United States [4,8]. In San Diego, approximately 23% of parents reported that their child had seen a CAM provider in the past 12 month [3]. Parents who use CAM therapies are often accustomed to seeking medical treatment for their children. In the United States, large proportions of children who take herbal supplements also take prescriptions or over-the-counter medications concurrently [18]. Because CAM care can be sought for both sick and routine care [3], children with chronic illnesses are at least three times more likely to use CAM than healthy children [19].

CAM use among children has been reported in Hong Kong, Singapore and the United States [5,8,18,20-23]. In Taiwan, most studies reported TCM use patterns focused on the adult population [10,13-15,24-28]. However, limited studies have reported on TCM use patterns among children in Taiwan. The relationship between socioeconomic factors and TCM utilization among adults in Taiwan was investigated in previous researches [14,24-26,28,29]. The association between high socioeconomic status (SES) and TCM use was also found in adult cancer patients in Taiwan [26]. A study based on Taiwan's National Health Insurance also showed that high education and income were associated with TCM use among adults [25]. However, no study has demonstrated the relationship between SES and pediatric TCM use in Taiwan. This study used data from the Taiwan National Health Interview Survey (NHIS) to investigate the association between SES and TCM utilization among children.

## Methods

### Study Design and Participants

Before interviews were conducted, the interviewers explained the program to the parents/guardians of children and invited their participation. Informed (written) consents were then obtained from the parents/guardians of children. This study was approved by the Bureau of Health Promotion of Taiwan.

Taiwan has a population of approximately 23 million people distributed across 7 cities and 18 counties. In 2001 the National Health Research Institute and Bureau of Health Promotion of Taiwan conducted a nation-wide NHIS survey using a face-to-face interview questionnaire [14,30]. The 2001 NHIS included a representative sample of 22,121 interviewees from the non-institutional population. With a standardized face-to-face interview questionnaire, the NHIS used a multi-stage stratified sampling scheme to collect a representative sample of Taiwan's population. Approximately 323 interviewers were trained to administer these interviews. These interviewers explained the study's purpose at the beginning of each interview. If the interviewee was of eligible age, the interview was either initiated at that time or scheduled for later. The 2001 NHIS was a cross-sectional survey with sampling and measurement details similar to those described elsewhere [24]. The response rate for our survey was 94%.

### Data Collection

The 2001 NHIS content included questions about sociodemographic factors, health status, self-reported height and weight, medical services utilization, lifestyle and heath behaviors. The questionnaire included several questions on the use of medical services, including: (1) In the past month, excluding dental care, have you used AM outpatient services (e.g., routine prenatal checks, health examinations, hospitalizations or emergency room visits)? or (2) In the past month, have you been to any TCM hospitals or clinics? Individuals who reported AM use were defined as AM users and those reporting TCM use were defined as TCM users.

### Definition and Variables

TCM includes the following treatments: herbal medicine, acupuncture, moxibustion, bone reduction, traditional trauma treatment, traditional dislocation treatment, traditional fracture treatment, Tuina, Baguan and other therapies. Its practitioners are licensed TCM physicians and practice in a hospital or clinic. TCM is legal in Taiwan, and according to Taiwanese medical law, TCM physicians are allowed to advertise the medical benefits of TCM. At the end of 2001, there were 2 public and 42 private TCM hospitals in Taiwan as well as 2,544 private TCM clinics providing TCM ambulatory care [10].

Urbanization: There are 359 townships and city districts in Taiwan. We calculated the population density (persons/km^2^) for each of these administrative units by dividing the population (persons) of the unit by its area (km^2^). The first, second and third tertiles were considered to be areas of low, moderate and high urbanization, respectively [31]. We calculated the density of physicians per administrative unit using the number of physicians per 10,000 persons.

### Statistical Analysis

The SES of the head of the household was taken as the SES of each child. We calculated scores for the head of the household's education and occupation in an effort to calculate SES scores according to Kuppuswamy's SES scale [32]. This entailed summing the scores for education (profession or honors = 7, graduate or postgraduate = 6, intermediate or post-high school diploma = 5, high school certificate = 4, middle school certificate = 3, primary school certificate = 2, illiterate = 1), occupation (profession = 10, semi-profession = 6, clerical worker or shop owner or farmer = 5, skilled worker = 4, semi-skilled worker = 3, unskilled worker = 2, unemployed = 1) and income (New Taiwan Dollars [NTDs]: < 10,000 = 1, 10,000-19,999 = 2, 20000-39,999 = 3, 40000-59999 = 4, 60,000-79,999 = 6, 80000-99,999 = 10, > 100,000 = 12) to calculate an overall SES score [32]. Each head of household's occupation was assessed according to criteria from a local study of SES in Taiwan [33]. The SES scores were divided into the following quartiles: first (scores 3-9), second (scores 10-11), third (scores 12-14), and fourth (15-29 scores). In 2001, one US dollar was equal to 35 NTDs and one NTD was the equivalent of 1.47 Indian rupees.

We compared the children's mean ages, body mass index and number of TCM visits. Moreover, we compared the children with respect to their SES quartile scores according to the following criteria: mean age, mean SES score, and the mean number of children in the household. We used analysis of variance statistical methods to test whether these factors varied by SES scores. Chi-square tests were then used to compare children from different SES quartile scores with respect to the gender distribution, living in high-density urban areas, and use of TCM, as well as the use of AM.

Adjusted odds ratios (OR) and 95% confidence intervals (CI) were estimated for the relationship between TCM use and SES scores using four different logistic regression models. Model 1 employed a multivariate logistic regression to calculate a crude OR, adjusting for the sex and age of the children. Model 2 adjusted for body mass index, children's use of AM and head of household's smoking and alcohol consumption. Model 3 adjusted for the covariates in model 2 as well as urbanization. Model 4 adjusted for the covariates in model 3 and for the head of household's use of TCM. Further stratified analyses attempted to clarify how TCM use was associated with SES scores separately for boys and girls. All analyses were performed using SAS software, version 8.0 (SAS Institute Inc., Carey, NC). Two-tailed probability values of < 0.05 were considered statistically significant.

## Results

This study found that 4.7% of the 5,971 eligible pediatric participants had used TCM within the past month. Compared to children from the lowest SES quartile, children in the highest SES quartile had higher average numbers of TCM visits (0.12 ± 0.64 vs. 0.09 ± 0.53, p < 0.0001), a higher prevalence of TCM usage (5.9% vs. 4.3%, p = 0.024) and greater use of AM (39.5% vs. 32.5%, p = 0.0005) (Table 1). Children from high SES families were also more likely to live in high-urban areas (27.2% vs. 14.8%, p < 0.0001). In contrast, the average age (8.5 ± 5.2 vs. 10.0 ± 5.2, p < 0.0001) and body mass index (17.8 ± 3.9 vs. 18.5 ± 4.5, p < 0.0001) were lower in children from the highest SES quartile than those from the lowest SES quartile. A U-shaped relationship was found between the prevalence of TCM use and the SES quartiles among this pediatric population.

**Table 1:** The characteristics of study pediatric population by socioeconomic status

	Socioeconomic status, scores				
		
	1st	2nd	3rd	4th	
	Mean ± SD	Mean ± SD	Mean ± SD	Mean ± SD	p-value
Number	1325	1370	1753	1523	
Children					
Age, years	10.0 ± 5.2	9.3 ± 5.1	8.9 ± 5.1	8.5 ± 5.2	< 0.0001
Body mass index, kg/m^2^	18.5 ± 4.5	18.3 ± 4.3	18.0 ± 4.0	17.8 ± 3.9	< 0.0001
Use of traditional Chinese medicine, visits	0.09 ± 0.53	0.06 ± 0.38	0.08 ± 0.41	0.12 ± 0.64	0.027
Boys, %	51.3	49.7	51.2	54.1	0.11
Live in high urbanization, %	14.8	14.4	16.6	27.2	< 0.0001
Use of traditional Chinese medicine, %	4.3	3.6	4.9	5.9	0.024
Use of allopathic medicine, %	32.5	36.4	38.6	39.5	0.0005
Household leaders					
Age, years	41.9 ± 11.1	38.0 ± 7.9	38.2 ± 7.4	39.2 ± 6.6	< 0.0001
Socioeconomic status, scores	7.5 ± 1.6	10.5 ± 0.5	12.8 ± 0.8	18.9 ± 3.4	< 0.0001
Occupation, scores	0.3 ± 0.5	0.9 ± 0.7	1.7 ± 0.9	2.6 ± 0.8	< 0.0001
Numbers of household leader's children	2.5 ± 1.1	2.5 ± 1.0	2.4 ± 1.0	2.3 ± 1.1	< 0.0001
Education ≥ 13 years, %	2.2	2.9	20.8	78.9	< 0.0001
Income 40,000-79.999 NTD, %	6.0	26.4	57.1	67.3	< 0.0001
Smoking, %	46.6	63.1	50.4	41.8	< 0.0001
Alcohol drinking, %	37.4	46.1	41.6	41.6	0.0001
Use of traditional Chinese medicine, %	8.6	8.8	9.1	6.1	0.008

SD, standard deviation; TCM, traditional Chinese medicine.

Heads of households from the highest SES quartile were older (39.2 ± 6.6 vs. 41.9 ± 11.1, p < 0.0001) and had more children (2.3 ± 1.1 vs. 2.5 ± 1.1, p < 0.0001) compared with those from the lowest quartiles. Among the heads of households, significant differences in smoking (p < 0.0001), alcohol consumption (p < 0.0001), and TCM use (p < 0.0001) were also found between the SES quartiles.

In the multivariate logistic regression (Table 2), the adjusted OR for TCM use was higher among children from the fourth SES quartile than among children in the first SES quartile (see model 1). The adjusted ORs in models 2, 3, and 4 were 1.43 (95% CI 0.99-2.06), 1.43 (95% CI 0.98-2.08), and 1.49 (95% CI 1.02-2.17), respectively. A significant p value for the OR trend was found in each model.

**Table 2:** Odds ratios and 95% confidence intervals of use of traditional Chinese medicine among children in association with socioeconomic status

	Socioeconomic status, scores								
		
	1st		2nd		3rd		4th		
	OR	(95% CI)	OR	(95% CI)	OR	(95% CI)	OR	(95% CI)	p for Trend
Model 1	1.00	(reference)	0.84	(0.57-1.24)	1.18	(0.84-1.66)	1.45	(1.03-2.04)	0.007
Model 2	1.00	(reference)	0.77	(0.50-1.19)	1.12	(0.77-1.62)	1.43	(0.99-2.06)	0.013
Model 3	1.00	(reference)	0.77	(0.50-1.19)	1.12	(0.77-1.63)	1.43	(0.98-2.08)	0.015
Model 4	1.00	(reference)	0.76	(0.49-1.17)	1.11	(0.76-1.61)	1.49	(1.02-2.17)	0.009

CI, confidence interval; OR, odds ratio.

Model 1: adjusted pediatric sex and age.

Model 2: adjusted pediatric sex, age, body mass index, use of allopathic medicine, household leader's smoking, alcohol drinking.

Model 3: adjusted covariates in model 2 plus urbanization.

Model 4: adjusted covariates in model 3 plus household leader's use of traditional Chinese medicine.

**Figure 1:**
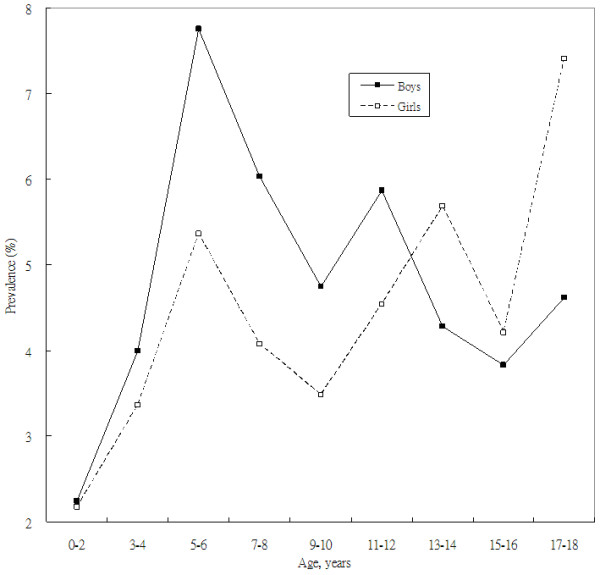
**Prevalence of traditional Chinese medicine utilization among the pediatric population by sex and age**.

In the sex-stratified analysis (Table 3), there was no association between SES and TCM use among boys. However, among girls, the ORs for the relationship between TCM use and SES were 2.02 (95% CI 1.20-3.39) in model 1, 1.82 (95% CI 1.14-3.38) in model 2, and 2.17 (95% CI 1.24-3.78) in model 4. The U-shaped pattern between SES and TCM use was investigated only for girls. As shown in Figure 1, the prevalence of TCM use was 2.2% in girls between 0-2 years of age, 5.4% in girls between 5-6 years of age, 3.5% in girls between 9-10 years of age, and 7.4% in girls 17-18 years of age. The age-stratified analysis in model 1 (Table 4) showed that compared to girls in the lowest SES quartile, girls in the highest SES quartile had a higher OR for TCM use (OR 1.95; 95% CI 1.04-3.67). In model 3 the corresponding OR was 2.10 (95% CI 1.08-4.08). Compared to girls in the first SES quartile, girls in the fourth SES quartile had the highest OR for TCM use (OR 2.47; 95% CI 1.25-4.90). The U-shape relationship between SES and TCM use existed in girls aged 10-18 years. With per 5 SES scores increase, the OR for TCM use was 1.69 (95% CI 1.32-2.16).

**Table 3:** Odds ratios and 95% confidence intervals of use of traditional Chinese medicine among children in association with socioeconomic status by sex

	Socioeconomic status, scores								
		
	1st		2nd		3rd		4th		
	OR	(95% CI)	OR	(95% CI)	OR	(95% CI)	OR	(95% CI)	P for Trend
Boys									
Model 1	1.00	(reference)	0.91	(0.55-1.49)	0.93	(0.59-1.49)	1.10	(0.69-1.74)	0.65
Model 2	1.00	(reference)	0.82	(0.47-1.45)	0.98	(0.59-1.62)	1.13	(0.68-1.87)	0.49
Model 3	1.00	(reference)	0.82	(0.46-1.44)	0.96	(0.58-1.59)	1.04	(0.62-1.75)	0.71
Model 4	1.00	(reference)	0.80	(0.45-1.41)	0.95	(0.57-1.59)	1.07	(0.63-1.79)	0.64
Girls									
Model 1	1.00	(reference)	0.76	(0.41-1.42)	1.57	(0.94-2.64)	2.02	(1.20-3.39)	0.0006
Model 2	1.00	(reference)	0.70	(0.36-1.36)	1.29	(0.75-2.23)	1.82	(1.06-3.10)	0.005
Model 3	1.00	(reference)	0.69	(0.36-1.34)	1.31	(0.76-2.25)	1.96	(1.14-3.38)	0.002
Model 4	1.00	(reference)	0.69	(0.35-1.35)	1.29	(0.74-2.24)	2.17	(1.24-3.78)	0.001

Abbreviations: CI, confidence interval; OR, odds ratio.

Model 1: adjusted pediatric sex and age.

Model 2: adjusted pediatric sex, age, body mass index, use of allopathic medicine, household leader's smoking, alcohol drinking.

Model 3: adjusted covariates in model 2 plus urbanization.

Model 4: adjusted covariates in model 3 plus household leader's use of traditional Chinese medicine.

**Table 4:** Odds ratios and 95% confidence intervals of use of traditional Chinese medicine among children in association with household leaders' socioeconomic status among girls by age

	Socioeconomic Status, Scores										
				
	1st		2nd		3rd		4th			Per 5 Scores Increase	
	OR	(95% CI)	OR	(95% CI)	OR	(95% CI)	OR	(95% CI)	P for Trend	OR	(95% CI)
Age, years											
0-9											
Model 1	1.00	(reference)	1.16	(0.41-3.30)	1.92	(0.76-4.86)	2.20	(0.87-5.59)	0.041	1.28	(0.96-1.72)
Model 2	1.00	(reference)	0.95	(0.32-2.82)	0.94	(0.35-2.55)	1.48	(0.57-3.85)	0.34	1.12	(0.79-1.58)
Model 3	1.00	(reference)	0.92	(0.31-2.74)	0.95	(0.35-2.56)	1.53	(0.58-4.01)	0.30	1.14	(0.80-1.62)
Model 4	1.00	(reference)	0.82	(0.27-2.48)	0.88	(0.32-2.39)	1.49	(0.57-3.94)	0.29	1.14	(0.80-1.62)
10-18											
Model 1	1.00	(reference)	0.57	(0.25-1.30)	1.38	(0.73-2.60)	1.95	(1.04-3.67)	0.007	1.51	(1.21-1.88)
Model 2	1.00	(reference)	0.52	(0.22-1.23)	1.34	(0.70-2.59)	1.87	(0.98-3.57)	0.011	1.50	(1.20-1.88)
Model 3	1.00	(reference)	0.51	(0.21-1.21)	1.37	(0.71-2.64)	2.10	(1.08-4.08)	0.005	1.61	(1.27-2.05)
Model 4	1.00	(reference)	0.53	(0.22-1.28)	1.39	(0.71-2.72)	2.47	(1.25-4.90)	0.002	1.69	(1.32-2.16)

Abbreviations: CI, confidence interval; OR, odds ratio.

Model 1: adjusted pediatric sex and age.

Model 2: adjusted pediatric sex, age, body mass index, use of allopathic medicine, household leader's smoking, alcohol drinking.

Model 3: adjusted covariates in model 2 plus urbanization.

Model 4: adjusted covariates in model 3 plus householder's use of traditional Chinese medicine.

## Discussion

This study was designed to provide a comprehensive analysis of the influence of SES on TCM utilization among children. The results of this large-scale NHIS study found that, among children in Taiwan, a higher prevalence of TCM visits was associated with higher SES. High-SES adolescent girls were more likely to visit TCM practitioners than low-SES girls. A significant trend was found between SES and TCM utilization, even after controlling for many of the relevant associated factors. To the best of our knowledge, this study is the first to examine the association between SES and TCM utilization.

Many studies have reported that high SES is associated with the use of medical health services [15,25,26,34,35]. Higher income levels and the presence of private insurance were associated with more TCM or AM consultations among people in Hong Kong [35]. In addition, the association between high SES and TCM use was also found in cancer patients in Taiwan [26]. Daly et al reported that both high education and high income were associated with TCM use among Caucasian adults in Taiwan [15]. A study based on Taiwan's National Health Insurance also showed similar conditions in Taiwan [25]. At present, no study has reported an association between SES and the use of unconventional medical services such as CAM or TCM in children. Although Shih et al [14] found that CAM use in Taiwan varied with respect to socioeconomic factors; they did not confirm an association between SES and TCM use. However, in this study we found that children of high-SES households were more likely to use TCM compared with children of low-SES households. In Taiwan, parental socioeconomic factors are meaningfully related to children's mental and physical health [36].

Because people of lower SES have greater morbidity, higher mortality and higher barriers to access to more advanced medical services, they tend to seek cheaper health services that are covered by public health insurance [34]. General health care and public health care are considered to be cheaper health care options that people of low SES are more likely to utilize. Yu et al [34] found that in Hong Kong, socioeconomic deprivation was associated with public health care use. Among patients with osteoarthritis in Hong Kong, low education and SES were associated with greater disease severity [31]. In general, socioeconomically disadvantaged populations experience inferior mental and overall health. Moreover, they report health service needs similar to or even greater than those of high-SES populations [37].

In Taiwan, TCM has frequently been used to treat diseases of the respiratory system, musculoskeletal disorders, injury and poisoning, and signs, symptoms and ill-defined conditions [25]. Menstrual discomfort is also a frequent reason for females to seek TCM treatment in Taiwan [38-41]. This study found that compared to low-SES girls, high-SES girls were more likely to seek TCM. In addition, further analysis found that girls between the ages of 10-18 years were more likely to visit a TCM practitioner compared to girls aged between 0-10 years. It has been documented that menarche can begin at 10 years of age (average = 13, 95% confidence interval = 11-15 years) in Taiwanese girls [42]. Wu found that the average age at menarche for adolescent girls in Taiwan was 12.11 years (95% confidence interval = 10.07-14.15 years) [43]. Consequently, we assumed that possibly these girls used TCM for treating menstrual problems [38-41]. High-SES adolescent girls were found more likely to seek TCM compared low-SES adolescent girls. Thus, we assumed that perhaps high-SES parents were capable of paying more for care for their adolescent girls' menstrual problems. National Health Insurance in Taiwan covers AM and TCM services. However, the types of Chinese herbal medicine covered by the NHI are limited to extracted TCM powder preparations prescribed by TCM physicians. Patients are required to pay out of their own pocket for crude drugs and other TCM products produced according to traditional methods. For example, the decoction method (boiling several prescribed crude drugs down to make a Chinese medicinal soup) has been used for thousands of years [29]. For females with menstrual problems, TCM is one of the choices. TCM treatments for menstruation problems included consultation, acupuncture, moxibution and herbal medicine, all covered in Taiwan's National Health Insurance. The four-agent decoction (i.e., Si Wu Tang) therapy [38] and other TCM herbal medicine formulas [44] demonstrate adequate effectiveness in reducing the menstrual pain associated with primary dysmenorrhoea. TCM in Taiwan, as in other countries, is not the principal source of medical care; however, the market for and effectiveness of TCM should not be ignored [45]. Because of the association between age at menarche and body mass index later in life, we adjusted the final model for children's body mass index [46].

Parents are care givers and guardians for children. They are also the decision makers for children's medicine-seeking behaviour. In Taiwan, medical doctors were considered a population with high SES. A recent local study showed interesting results that medical doctors with experience with TCM training had higher use of TCM services, as well as their relatives [27]. We considered that knowledge, attitude, and practice for parents have great impact on children's medicine-seeking behaviour. The concern about the health of children by parents pushes them to seek other choices for medical treatment and may be an important factor associated with TCM use in children. The high rate of CAM or TCM utilization among children is a source of increasing concern among pediatricians in Hong Kong and Singapore [22,23]. In Taiwan, limited information is available regarding the patterns and utilization of TCM among children. Our study is the first study to investigate the relationship between SES and TCM use among children in Taiwan.

CAM use varies by sex, race, geographic region, health insurance status, smoking habits and alcohol consumption [3]. CAM use by parents/caretakers is the best predictor for CAM use among children [4]. In this study's final model, we investigated how parents' SES influenced the TCM utilization by children, adjusting for the parents' own use of TCM. Because of the association between childhood obesity and SES reported in previous research, we also adjusted for childhood body mass index [47]. In Taiwan, people with low income, severe diseases, pregnancy, veteran, or some important occupational diseases and injuries are remitted a copayment for National Health Insurance. In this study, people with low income were included in the first quartile of income. Because the medical copayment remission, low-income people might have less barrier to medical economics compared with people of moderate income. In addition low-SES people have more morbidities than the general population. These reasons may explain why the U-shaped relationship exists between SES and TCM use among children in this study.

The principal strength of this study lies in its use of a large, nationally representative survey of a non-institutionalized pediatric population in Taiwan. However, this study still has several limitations. First, the responses to questions about children's TCM use are dependent on the parents' willingness to report such use accurately. Second, the results will also be biased if respondents misreport their SES. Finally, because this is a cross-sectional study, we cannot determine for certain whether parents' SES is the real cause of TCM use in children.

## Conclusion

Children from high-SES families were more likely to use TCM compared with children from low-SES families, especially among adolescent girls aged 10-18 years. We assumed that adolescent girls (10-18 years) visited TCM settings due to their menstrual problems. We also assumed high-SES parents to be capable of paying more money for care for their adolescent daughters' menstrual problems and thus more likely to visit TCM settings. Because of the high rate of TCM and CAM use among the Chinese populations in Canada and the United States [16,29], it is important that pediatricians be informed about such treatments and their popularity. CAM is an aspect of children's health care that should not be ignored [48]. By being aware of these alternative medical practices, physicians will be able to discuss CAM with parents, ensuring the continuity of essential conventional treatments. Further research is needed to better understand the nature of this finding and how it influences health outcomes.

## Abbreviations

AM: allopathic medicine; CAM: complementary and alternative medicine; NHIS: National Health Interview Survey; SES: socioeconomic status; TCM: traditional Chinese medicine.

## Competing interests

The authors declare that they have no competing interests.

## Authors' contributions

CCS, CCL, TFY, YCS and JGL were involved in the study concept, design, research questions, data interpretation and data acquisition. CCS CCL JGL contributed to data analysis and drafted the manuscript. All authors revised the article for intellectual content and approved the final version.

## Pre-publication history

The pre-publication history for this paper can be accessed here:

http://www.biomedcentral.com/1472-6963/12/27/prepub
